# In vivo imaging of choroidal angiogenesis using fluorescence-labeled cationic liposomes

**Published:** 2012-04-26

**Authors:** Jing Hua, Nikolai Gross, Brita Schulze, Uwe Michaelis, Hermann Bohnenkamp, Eric Guenzi, Lutz L. Hansen, Gottfried Martin, Hansjürgen T. Agostini

**Affiliations:** 1 University Eye Hospital, Albert-Ludwigs University of Freiburg, Freiburg im Breisgau, Germany; 2 MediGene AG, Lochhamer, Martinsried, Germany

## Abstract

**Purpose:**

Precise monitoring of active angiogenesis in neovascular eye diseases such as age-related macular degeneration (AMD) enables sensitive use of antiangiogenic drugs and reduces adverse side effects. So far, no in vivo imaging methods are available to specifically label active angiogenesis. Here, we report such a technique using fluorophore-labeled cationic liposomes (CL) detected with a standard clinical in vivo scanning laser ophthalmoscope (SLO).

**Methods:**

C57Bl/6 mice underwent laser coagulations at day 0 (d0) to induce choroidal neovascularization (CNV). Liposomes labeled with Oregon green, rhodamine (Rh), or indocyanine green (ICG) were injected into the tail vein at various time points after laser coagulation, and their fluorescence was observed in vivo 60 min later using an SLO, or afterwards in choroidal flatmounts or cryosections.

**Results:**

SLO detected accumulated fluorescence only in active CNV lesions with insignificant background noise. The best signal was obtained with CL-ICG. Choroidal flatmounts and cryosections of the eye confirmed the location of retained CL in CNV lesions. Neutral liposomes, in contrast, showed no accumulation.

**Conclusions:**

These results establish fluorophore-labeled CL as high affinity markers to selectively stain active CNV. This novel, non-invasive SLO imaging technique could improve risk assessment and indication for current intraocular antiangiogenic drugs in neovascular eye diseases, as well as monitor therapeutic outcomes. Labeling of angiogenic vessels using CL can be of interest not only for functional imaging in ophthalmology but also for other conditions where localization of active angiogenesis is desirable.

## Introduction

Age-related macular degeneration (AMD) is the leading cause of blindness in the Western world with a prevalence of 1.5% in the population 40 years or older (data for the United States). The incidence increases with age, affecting 12% of people over the age of 80 [1]. The number of patients having AMD is estimated to double by 2020, due to increased life expectancy [1]. Therefore, preventing and treating AMD play crucial roles in an individual patient’s quality of life as well as for the socioeconomic aspects of a sustainable health-care system [2,3]. AMD presents in two major forms: the exudative or wet form is characterized by activation of pathologic subretinal angiogenesis resulting in the formation of choroidal neovascularization (CNV) while the dry form presents with degeneration of the choroidal capillaries and/or retinal pigment epithelium (RPE). The exudative form with active CNV formation usually represents a rapidly progressive disease, and patients often develop severe visual impairment within months if left untreated [2].

Current treatments for wet AMD with angioinhibitory drugs such as bevacizumab or ranibizumab have demonstrated efficient reduction of subretinal fluid and maintenance of visual acuity [4]. Many other antiangiogenic agents are currently under investigation or in clinical trials. However, long-term repeated intraocular injections are required with all current drugs to maintain visual acuity. But accurate use of the treatments is critical to improve patient safety. Not only specific and quantitative detection of angiogenic activity in AMD is crucial to precisely define the need and time for intraocular reinjections of angioinhibitory drugs [5,6], but also sensitive diagnostic measures are needed to detect early stages of exudative AMD to initiate treatment at the first sign of angiogenic activation [7].

The standard imaging method for patients with wet AMD to date is fundus fluorescence angiography (FFA). FFA provides information about vessel perfusion and leakage, but not direct evidence of the proliferative activity of lesions. Therefore, the interpretation of FFA results to determine the activity of the lesion depends on individuals’ experience and subjective opinions. Detecting early onset of non-leaky CNV lesions is a challenging task using FFA. The interpretation of FFA results of minimally active CNV can be hampered, for example, by drusen that absorb fluorescein seen in the late phase of FFA. Loss of RPE can lead to hyperfluorescence in the early phase of FFA. In some large eye care centers, optical coherent tomography (OCT) in the time or spectral domain modus has thus developed to form one of the cornerstones of modern macular imaging and is sensitive in measuring retinal thickness and intra- or subretinal fluid. Combining OCT and FFA allows precise assessment of retinal thickness, retinal fluid accumulation, and fluorescein leakage, but neither method provides direct evidence of the angiogenic activity in CNV lesions. Nevertheless, FFA and OCT can be potentially further developed to image cellular and molecular processes using appropriate tracers and contrast agents.

Cationic liposomes (CL) exhibit high affinity for binding to sites of active angiogenesis in tumors, inflammation, and other sites of angiogenesis without extravasation [8]. CL are rapidly cleared from the circulation by the liver, spleen, and lung after intravenous injection [9-11]. They have been evaluated as potential drug-delivery vehicles for angiogenic tumors in animal models [12-18].

Based on the angiogenic nature of CNV lesions of wet AMD, we assessed the potential of CL as a delivery system for fluorophores in functional fundus imaging of active CNV. We synthesized CL with stable conjugation with Oregon Green 488 (OG) or indocyanine green (ICG), which are detectable in commonly used scanning laser ophthalmoscope (SLO) systems or fundus cameras used in FFA. The specific binding and clear detection of fluorophores in the active CNV lesions in a murine model suggest that our formulations of CL can be potentially used in patients with AMD to determine CNV activity precisely.

## Methods

A novel in vivo imaging method for activated angiogenesis sites in CNV lesions using fluorophore-labeled CL was established. OG, a fluorescein derivative with more favorable fluorescence properties, ICG, and lissamine-rhodamine B (Rh) were used as fluorescent labels. ICG and OG are detectable with commercially available SLO or other fundus cameras used in FFA.

### Synthesis and analytical characterization of fluorophore-labeled cationic liposomes

1,2 Dioleoyl-3-trimethylammonium propane (DOTAP) was from Merck Eprova (Schaffhausen, Switzerland), and 1,2 dioleoyl-sn-glycero-3-phosphocholine (DOPC) and 1,2 dioleoyl-sn-glycero-3-phosphoethanolamine-N-(lissamine rhodamine B sulfonyl) (Rh-DOPE) for making CL-Rh were from Avanti Polar Lipids, Inc. (Alabaster, AL). (Oregon Green 488) 1,2 dihexadecanoyl-sn-glycero-3-phosphoethanolamine (OG-DHPE) for making CL-OG was obtained from Invitrogen (Karlsruhe, Germany). Indocyanine green-1,2 di (cis-9-octadecenoyl)-sn-glycero-3-phosphoethanolamine (ICG-DOPE) for making CL-ICG was synthesized by Molecular Probes (Invitrogen, Karlsruhe, Germany, custom synthesis). Liposomes with a total lipid concentration of 10 mM were prepared by the lipid film method (see Table 1 for compositions). To prepare the liposomes, the respective lipids and lipid-coupled dyes were dissolved in 15 ml chloroform in a round bottom flask. The mixture was gently warmed to 40 °C, and the solvent was evaporated at reduced pressure in a rotary evaporator. The resulting lipid film was dried under vacuum for about 60 min, and then it was resuspended in an aqueous solution containing 10% trehalose. The resulting suspension of the multilamellar vesicles was extruded (10 ml Lipex extruder; Northern Lipids Inc., Vancouver, BC, Canada) five times through a 0.2 µm polycarbonate membrane (GE Osmonics, Minnetonka, MN), yielding unilamellar liposomes. To prepare 10 ml of neutral liposomes, 0.095 mmol DOPC and 0.005 mmol OG-DHPE were dissolved in chloroform and treated as described above.

**Table 1:** Summary of liposomal formulations.

		**Composition**		
**Formulation**	**Particle size/polydispersity index**	**DOTAP**	**DOPC**	**lipid coupled dye**
CL-OG	133 nm / 0.163	6 mM	3.5 mM	0.5 mM
CL-Rh	158 nm / 0.183	5 mM	4.5 mM	0.5 mM
CL-ICG	162 nm / 0.236	6 mM	3.5 mM	0.1 mM
neutral liposomes with OG	178 nm / 0.287	0 mM	9.5 mM	0.5 mM

Abbreviations: CL represents cationic liposomes, OG represents oregon green, ICG represents indocyanine green, DOTAP represents 1,2 Dioleoyl-3-trimethylammonium propane, and DOPC represents 1,2 dioleoyl-sn-glycero-3-phosphocholine.

The particle size and size distribution of the liposomal suspensions were analyzed with photon correlation spectroscopy (Malvern Zetasizer 3000; Malvern Instruments, Herrenberg, Germany), and the results are listed in Table 1. The concentration and purity of the liposomal components were checked with high-performance liquid chromatography (HPLC)-ultraviolet/visible spectroscopy.

The fluorescence properties of all formulations were optimized and checked with fluorescence spectroscopy using a fluorescence spectrophotometer (HORIBA Jobin Yvon GmbH, Munich, Germany). Liposomal formulations were diluted 1:50 (CL-ICG) or 1:2,000 (CL-OG) in trehalose. The excitation wavelength was set to 805±5 nm (ICG) or 490±5 nm (OG), and the emission was measured at 825±5 nm (ICG) or 534±5 nm (OG).

Mouse RPE was isolated from a freshly prepared posterior eye cup after the retina was removed and appropriately diluted. The absorption spectrum demonstrates the amount of light absorbed by RPE in different wavelengths and thus estimates the reduction in fluorescence emitted by various fluorophores, when they are located behind the RPE in vivo.

### Laser-induced choroidal neovascularization model

All animal procedures adhered to the animal care guidelines of the Institute for Laboratory Animal Research (Guide for the Care and Use of Laboratory Animals) in accordance with the Association for Research in Vision and Ophthalmology Statement for the Use of Animals in Ophthalmic and Vision Research and were approved by the local animal welfare committee.

The laser-induced CNV model in mice was performed as previously described [19]. Briefly, C57BL/6J mice (Charles River Laboratories, Hamburg, Germany) were anesthetized with an intraperitoneal injection of a ketamine (100 mg/kg) and xylazine (5 mg/kg) mixture. Then 0.5% tropicamide (Pharma Stulln, Stulln, Germany) and 0.5% phenylephrine (Ursapharm, Saarbrücken, Germany) eye drops were used to dilate pupils. Three laser spots per eye were induced with an argon laser (100 ms, 100 μm, and 150 mV, 532 nm, Visulas 532s, Carl Zeiss Meditec AG, Jena, Germany). At least three mice were used per time point and treatment.

### In vivo imaging with scanning laser ophthalmoscope

Fluorophore-labeled liposomes were injected into the tail vein (10 mM, 150 μl per mouse). Mice were anesthesized, and the pupils dilated as described above for CNV induction by laser. CNV were localized in the infrared mode of a digital SLO (HRA1, Heidelberg Engineering, Heidelberg, Germany). Fundus images were taken with filter sets for fluorescein (488 nm excitation, 530 nm emission) and ICG (795 nm excitation, 830 nm emission). Images were taken before and at different time points after intravenous injections of liposomal formulations and analyzed with ImageJ. The fluorescence intensity of a CNV (I_L_) was calculated by selecting a region of interest covering the laser CNV and dividing the sum of the intensity values of each pixel in that area by the number of the pixels. In the same way, background fluorescence (I_B_) was averaged from three areas of identical size and shape as the CNV area that were selected in close proximity to the CNV. An accumulation index was defined as I_L_ divided by I_B_. Values were tested for statistical significance with ANOVA, and differences were assumed to be significant when p<0.05.

### Sclerochoroidal flatmounts and microscopy

Immediately after the in vivo SLO images were taken, eyes were fixed in 4% formalin buffered with PBS (137 mM NaCl, 2.7 mM KCl, 10 mM Na_2_HPO_4_, 2 mM KH_2_PO_4_). After the anterior eye part and the retina were removed, the sclera and choroid complex including the RPE was flatmounted and examined with epifluorescence microscopy with 480 nm excitation and 510 nm emission filters without additional staining. Since ICG signals are detectable only in the infrared channel, which is unavailable in microscopes, only CL-OG injected eyes were included. Images were taken, and the selectivity in CNV lesions was quantified in the same way as in the SLO images. Three control areas were selected in healthy RPE outside the CNV area.

### Histological localization of cationic liposome and fluorescein isothiocyanate-lectin

Mice with L-CNV received an intravenous application of cationic liposomes labeled with rhodamine (CL-Rh) at d5 or d14 after laser coagulation (4 µl/g). The liposomes were allowed to circulate for 60 min before the animals received 100 µl fluorescein isothiocyanate (FITC) lectin (2 mg/ml, Vector Labs, Burlingame, CA) intravenously. After 5 min, the animals were perfused with 1% paraformaldehyde. Eyes were removed and embedded in optimum control temperature medium (TissueTek, Sakura, Japan) and quick-frozen in a Leica cryostat (Leica, Wetzlar, Germany). Then 50 µm sections were cut, dried for 30 min at room temperature, fixed in 4% buffered formalin, and stained with 10 µM ToPro-3 (Invitrogen, Karlsruhe, Germany) for 1 min at room temperature. After being washed with PBS, sections were mounted in Dako faramount aqueous (Dako, Hamburg, Germany), left to dry for 60 min at room temperature in the dark, and analyzed with a Leica TCS SP2 confocal microscope (Leica, Heidelberg, Germany) at excitation wavelengths of 488 nm, 543 nm, and 633 nm.

## Results

### Optimization of fluorophore-labeled liposomes

**Figure 1:**
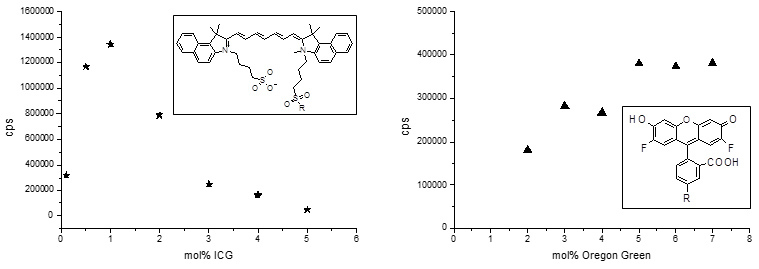
Cationic liposomes (CL) formulations labeled with indocyanine green (ICG) or Oregon green (OG) were internally quenched. Liposomes composed of 60 mol% 1,2 dioleoyl-3-trimethylammonium propane (DOTAP), 40 - x mol% 1,2 dioleoyl-sn-glycero-3-phosphocholine (DOPC), and x mol% ICG-DOPE or OG-DOPE were prepared and diluted 1:50 (CL-ICG) or 1:2,000 (CL-OG). Intraliposomal quenching reduces the fluorescence in the liposomal membrane at concentrations of more than 0.5 mol% ICG or 5 mol% OG. The fluorescence signal of ICG is ten times lower than that of OG if the different dilutions are taken into account.

The fluorophores OG, ICG, and Rh were stably linked to CL as they were covalently bound to phospholipids. This was confirmed with high-performance liquid chromatography analysis that did not detect any unbound fluorophore at any time during the study. Fluorescence intensity was optimal at 1 mol% ICG and 5 mol% OG in total liposomes due to intraliposomal quenching at higher fluorophore ratios (Figure 1). The inset in Figure 1 shows the respective molecular structure; OG differs from fluorescein only by having two F atoms at the aromatic ring system. In both structures, “R” indicates where the linker to the lipid moiety has been attached. Quenching of Rh was similar to OG in CL. Therefore, Rh was also used at a concentration of 5 mol%.

### In vivo short-term kinetics of fluorophore-labeled liposomes (scanning laser ophthalmoscope imaging)

**Figure 2:**
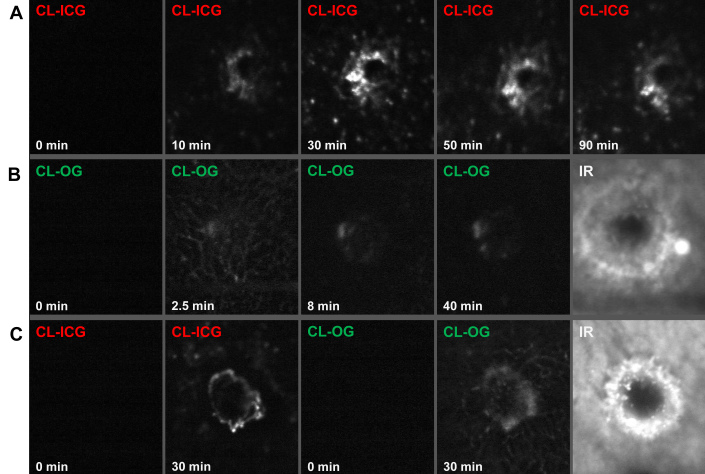
Kinetics of accumulation of cationic liposomes (CL)- indocyanine green (ICG) or CL-Oregon green (OG) was observed with the SLO in vivo. **A**: ICG-CL were injected at d14, and scanning laser ophthalmoscope (SLO) images were recorded in a single choroidal neovascularization (CNV). While images taken before injection of ICG (0 min) showed no signal, the ICG fluorescence (795 nm excitation, 830 nm emission) became detectable in the CNV after 10 min. Maximal intensity was observed between 30 min and 90 min with a slow decrease afterwards. Other parts of the fundus were not specifically stained. **B**: OG-CL were injected at d10, and SLO images were recorded in a single CNV. This series shows the early signal in the capillaries 2–3 min after injection that disappears quickly [8] while the OG-CL signal in the CNV is coming up later and for a longer time as shown in **A**. **C**: ICG-CL (40%) and CL-OG were mixed and injected at d14, and SLO images were recorded in a single CNV. Note that the OG signal is somewhat weaker. IR: CNV lesion in infrared modus.

**Figure 3:**
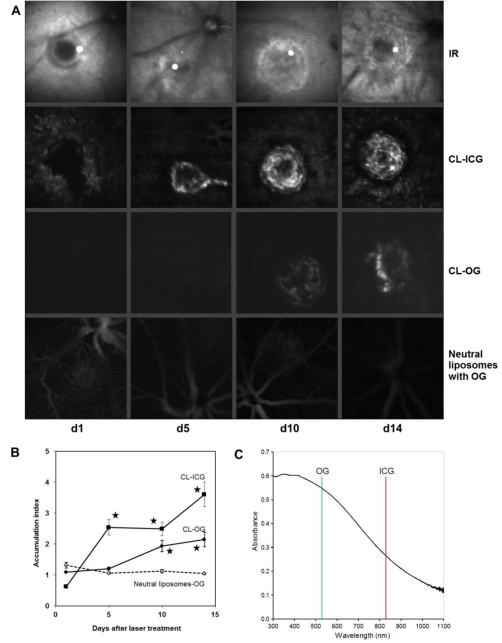
Accumulation of cationic and neutral liposomes was observed with the scanning laser ophthalmoscope (SLO) in vivo. Cationic liposomes (CL)-indocyanine green (ICG), CL-Oregon green (OG), or neutral liposomes labeled with OG were applied intravenously at d1, d5, d10, or d14 after laser treatment. SLO images were recorded for each laser choroidal neovascularization (CNV) 60 min later. **A**: One representative CNV is shown for each time point and for each of the formulations tested. The CL-ICG images are taken from the same CNV in one mouse, and the corresponding infrared (IR) images are shown for comparison. The other images are taken from different animals. Although neutral liposomes did not accumulate within the CNV, CL-OG was found from d10 onwards and CL-ICG starting from d5. **B**: The ratio of accumulation of liposomes in the CNV to the control area was calculated for each CNV as described in the methods section. The means and the standard errors of the mean are shown. Values for CL-OG at d10 and d14 and for CL-ICG at d5, d10, and d14 were significantly higher compared to those of neutral liposomes. Data are means obtained from five mice. Error bars indicate SEM, and asterisks indicate statistical significance (p<0.05 as compared to d1). **C**: Absorption spectrum of mouse RPE. After the retina as removed, the RPE was scraped out, homogenized by pipetting, and diluted in water. Transmission is higher at longer wavelengths. The OG emission was detected at 530 nm and that of ICG at 830 nm. The murine RPE absorption was about twice as high at 530 nm as at 830 nm. This gives an estimate of the reduction of fluorescence emitted by fluorophores when they are located behind the RPE in vivo and is one of the reasons why ICG is more suitable for diagnostics of sub-RPE lesions than OG or fluorescein.

The distribution of CL labeled with ICG or OG in the fundus was followed by SLO after intravenous injection. Figure 2 shows representative images at various time points after application of labeled CL-ICG or CL-OG 14 days after laser coagulation. No specific signal was detected before CL-ICG or CL-OG was injected. Circulation of CL formulations in the blood plasma was faintly visible for 2–3 min after application based on the respective fluorescence signal (Figure 2B). After that, fast accumulation of fluorescence at the CNV site was observed. Typically, the signal due to CL-OG was visible between 20 and 120 min after application. The CL-ICG signal was stronger and therefore was visible as early as 5 min after application and was observed for several hours, in some cases even for 24 h. The CL-ICG and CL-OG peak signal was observed between 30 and 90 min so that a measuring time point of 60 min after injection was chosen in the following experiments. CL-ICG resulted in a more intense and precise image compared to CL-OG (Figure 2C). In contrast, neutral liposomes did not accumulate at the laser site but remained much longer in circulation (Figure 3) similar to ICG or fluorescein in angiography.

### Kinetics of choroidal neovascularization formation detected with scanning laser ophthalmoscope

CL-OG, CL-ICG, and neutral liposomes labeled with OG were intravenously applied to mice on various days after laser coagulation. SLO images were recorded 60 min after injection. The CL-ICG yielded a clear signal five days after laser treatment. Both CL formulations showed a significant enrichment at the CNV site at d10 and d14 compared to neutral liposomes (Figure 3). The highest value was reached by CL-ICG at d14, where a 3.6-fold accumulation in the CNV was found. The CL-OG showed a weaker signal than CL-ICG probably because of absorption of the OG fluorescence by the RPE (compare Figure 3C). This shows again that CL-ICG’s signal was superior to that of CL-OG.

In the d1 CL-ICG image (Figure 3), the central laser burn was a black hole surrounded by CL-ICG signal (accumulation <1) probably due to activation of endothelial cells through local inflammation. This hole was gradually filled in with blood vessels during the course of the study as illustrated in the CL-ICG images suggesting that the accumulation of CL correlates with the growth of choroidal neovessels within the CNV.

The SLO images obtained with CL represented the morphology of CNV lesions well at d10 and d14, which appeared not as a homogenous distribution but as hot spots whose structure resembled blood vessels (Figure 2 and Figure 3). Neutral liposomes showed no accumulation in the CNV lesions (Figure 3), but remained in the circulation even 60 min after the intravenous injection similar to ICG or fluorescein in angiography. This demonstrates that the cationic charge is important for the accumulation of CL, which depends on the biologic status of the growing CNV.

### Confirmation of laser-choroidal neovascularization development with flatmount analysis

**Figure 4:**
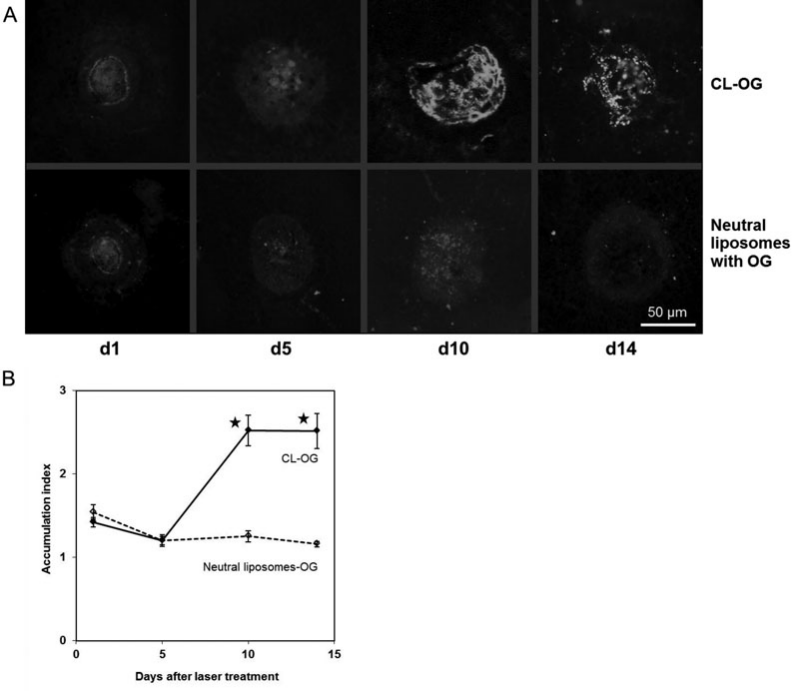
Accumulation of cationic and neutral liposomes was observed in flatmount preparations. After the scanning laser ophthalmoscope (SLO) images described in Figure 3 were taken, choroidal-scleral flatmounts were prepared from the mice treated with cationic liposomes (CL)-Oregon green (OG) or neutral liposomes labeled with OG. Images were taken using fluorescence microscopy. Representative ones are shown in **A**. They were evaluated the same way as the SLO images (**B**). Values for CL-OG at d10 and d14 were significantly higher compared to those of neutral liposomes. The results confirmed that the kinetics were the same as for the SLO images. Data are means obtained from five mice. Error bars indicate SEM, and asterisks indicate statistical significance (p<0.05 as compared to d1).

The choroids of the mice that were imaged with OG-labeled liposomes were flatmounted and investigated with fluorescence microscopy. Accumulation indices were computed analogous to the procedure with SLO and are shown in Figure 4B. Similar to the SLO data in Figure 3B, there was a significantly increased accumulation of CL-OG compared to neutral liposomes labeled with OG at d10 and d14 confirming that CL bound specifically to the CNV site. The CL-OG signal was much weaker at d1 and d5, accompanied by homogeneous background staining. Flatmounts from mice treated with neutral liposomes showed background staining as well. Background staining is typically derived from tissues such as muscle showing autofluorescence at the wavelength used for detecting OG.

### Histological localization of cationic liposomes

**Figure 5:**
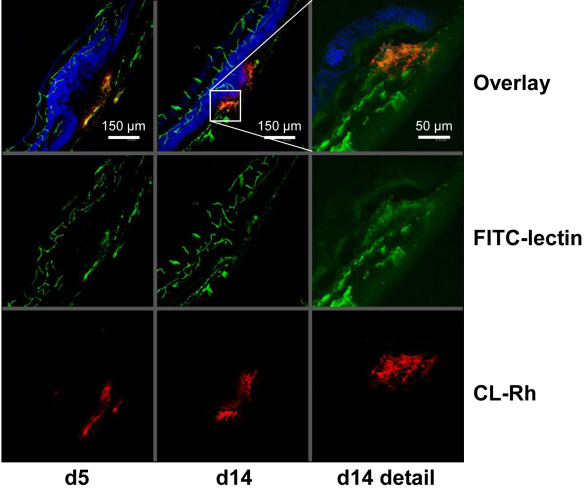
Cationic liposomes (CL) were localized in the choroidal neovascularization (CNV). At d5 or d14 after laser coagulation, CL labeled with rhodamine were applied intravenously. Sixty min later, the mice received FITC-lectin. Then, the eyes were enucleated, and cryosections were prepared. The CNV is situated between the retina and the choroid at the site where the nucleic layers (blue) of the retina are distorted. CL were found exclusively in the CNV.

To detect CL in histological sections, CL-Rh was applied intravenously, and blood vessels were labeled with fluorescein cyanate (FITC) lectin. Figure 5 shows representative images of laser-CNV at d5 and d14 after laser coagulation. The images show a well defined vessel structure in the retina and in the choroid. Staining with ToPro-3 allowed the nuclear layers of the retina that were deformed at the laser site to be identified. CL were exclusively found at the laser site. However, CL were not confined to the vessels but showed some spotty distribution around the vessels within the area of the CNV. There was no non-specific binding of CL to resting blood vessels, to photoreceptors, or to other structures. The amount of CNV labeled with CL-Rh increased from d5 to d14 confirming the results from the SLO and flatmount analysis.

## Discussion

The visualization of vascular cells involved in angiogenesis helps to determine the activity of a pathological process and to monitor the therapeutic success of angioinhibitory therapy, for example, in AMD, a disease affecting millions of people worldwide (1.75 million in the US [1]). The data presented here show that CL can be labeled with different diagnostic dyes, accumulate specifically at the site of experimentally induced choroidal neovascularization, and are detected with standard clinical scanning laser ophthalmoscopy in a CNV mouse model. Although angiography with fluorescein or ICG is based primarily on extravasation due to leaky blood vessels, imaging with CL allows sites with active angioproliferation to be visualized and could therefore prove to be a crucial step toward functional imaging of angiogenesis in ophthalmology.

The fluorescent dyes fluorescein and ICG are routinely used in fundus fluorescence angiography using SLO. In this study, CL were labeled with such dyes or their derivatives at low concentrations (1 and 5 mol% fluorescent dye) that did not affect the liposome’s capacity to bind to active angiogenesis sites. Other groups have also found this in animal models of cancer and chronic inflammation [8,13] as well as acute pulmonary inflammation [20]. Compared to standard fluorescence angiography, the fluorophore dose needed for imaging an angiogenetically active site with CL was about 0.5% of the fluorescein or ICG dose that would be applied in angiography.

The overall quality of the signal in the SLO was much better for CL-ICG than for CL-OG. In vitro, the fluorescence emission of the CL-ICG formulation was only about 10% of that measured for the CL-OG formulation (Figure 1). On the other hand, the laser intensity at 790 nm was about tenfold higher than that at 480 nm. In addition, the signal due to OG was strongly quenched by tissue (especially RPE) and blood components, so that ICG provided images with higher contrast than OG.

Previous studies reported the specific binding of cationic liposomes to angiogenic tumor blood vessels [8,13,14] and to areas of inflammation [8]. Similar results were found in the lung where CL-Rh was accumulated at sites stimulated by lipopolysaccharide [20]. Binding of CL to active angiogenesis sites has been reported to result in accumulation index values of about 3 (in vivo imaging) in tumor models [13]. Here, accumulation index values of about 2–4 are reported based on SLO imaging. Moreover, the SLO images presented here are of high quality and contrast as is expected for FFA.

The positive charge of CL is critical for preferential binding by or uptake into angiogenic endothelial cells [13]. In the present study, the preferential accumulation of different fluorophore-labeled CL formulations but not neutral liposomes at active angiogenesis sites in CNV was consistently found with in vivo SLO fundus imaging, epifluorescence microscopy of choroidal flatmounts, and fluorescence microcopy of cross-sections of the CNV lesions. The SLO images presented here (especially those with CL-ICG) demonstrate that the fluorescence signal was located at the edge of the lesion and grew from there. The signal was not uniformly distributed over the lesional site as typically observed in fluorescein angiography where leakage is the underlying mechanism. No extravasal diffusion of the dye was seen here. Instead, structures resembling blood vessels were identified. Accumulation of CL was closely associated with lectin-binding endothelial cells of vessels in cross sections (Figure 5), and the accumulation was strictly limited within the laser lesion. Overall, all imaging tools support the view that CL bind to active angiogenesis sites in the CNV.

The CL-ICG SLO images show some additional staining surrounding the laser spot. This may be attributed to cells involved in active angiogenesis similar to those PECAM1 (CD31) positive cells observed in rat CNV at d2 [21] indicating that CL-ICG detects early stages of angiogenesis.

In the laser-induced CNV mouse model, the maximal size of the CNV membrane is reached 14 days after laser treatment [19]. A similar time course was reported for monkeys [22]. In this study, the maximal signal of labeled CL was also observed at d14 showing that it correlated with the growth of the CNV membrane. Although CL-ICG was already detectable at d5, CL-OG was found starting at d10.

CL-OG and CL-ICG showed a broad intensity maximum at least between 30 and 90 min after application. This is a comfortable time window for thorough clinical evaluation of a patient, even if recording of both eyes is required. In addition, CL-ICG can be applied simultaneously with normal fluorescein angiography to characterize activation sites and correlate them with leakage phenomena in the same setting [23,24].

In humans, the same CL loaded with paclitaxel (EndoTAG-1) were used in a phase I study to treat tumors. They were generally well tolerated [25]. Some toxic effects such as fatigue or hypersensitivity reactions may be attributed to the paclitaxel load rather than to the CL. The fluorophores fluorescein and ICG used in the present study are commonly used in FFA and well tolerated, too. In addition, no toxic effects were observed in mice during this study.

In summary, the rationale for imaging using labeled CL is manifold: this method can help identify initial phases of choroidal neovascularization, and might serve to monitor the activity of choroidal angiogenesis, treatment success, or predict treatment outcome. It may help to gain a better understanding of the therapeutic effect of antivascular endothelial growth factor treatment and to predict when the disease will reappear by gaining information about the CNV activity in addition to size and leakage. Monitoring angiogenic activity can be of relevance not only in patients with wet AMD but also in patients with diabetic retinopathy or retinal vein occlusion at risk of developing proliferative retinopathy.
